# Results from the 2022 Mexican report card on physical activity for children and adolescents

**DOI:** 10.3389/fpubh.2023.1304719

**Published:** 2024-01-05

**Authors:** Gabriela Argumedo, Juan Ricardo López y Taylor, Julissa Ortiz Brunel, Alejandro Gaytán-González, Inés González-Casanova, Martín Francisco González Villalobos, Alejandra Jáuregui, Edtna Jáuregui Ulloa, Catalina Medina, Yoali Selene Pacheco Miranda, Marcela Pérez Rodríguez, Ricardo Alejandro Retano Pelayo, María del Pilar Rodríguez Martínez, Karla I. Galaviz

**Affiliations:** ^1^Centro de Investigación en Nutrición y Salud, Instituto Nacional de Salud Pública, Cuernavaca, Morelos, Mexico; ^2^Colegio de Ciencias y Humanidades, Universidad Nacional Autónoma de México, Mexico City, Mexico; ^3^Departamento de Ciencias del Movimiento Humano, Centro Universitario de Ciencias de la Salud, Universidad de Guadalajara, Guadalajara, Jalisco, Mexico; ^4^Physical Activity Promotion and Chronic Disease Prevention Unit, School of Kinesiology, The University of British Columbia, Vancouver, BC, Canada; ^5^Indiana University School of Public Health Bloomington, Bloomington, IN, United States; ^6^Centro de Adiestramiento en Investigación Clínica, División de Desarrollo de la Investigación, Instituto Mexicano del Seguro Social, Mexico City, Mexico; ^7^Coordinación de Cultura y Educación para un Estilo de Vida Saludable, Sistema de Educación Media superior, Universidad de Guadalajara, Guadalajara, Jalisco, Mexico; ^8^Instituto Tecnológico y de Estudios Superiores de Occidente (ITESO), Tlaquepaque, Jalisco, Mexico

**Keywords:** youth, sedentary behavior, active transportation, active play, Latin America

## Abstract

**Introduction:**

The Mexican Report Card on Physical Activity for Children and Adolescents aims to assess the prevalence of movement behaviors and opportunities to perform them.

**Methods:**

Data on 11 indicators were obtained from national health surveys, census data, government documents, websites, and published studies. Data were compared against established benchmarks, and a grade between 0 and 10 was assigned to each indicator.

**Results:**

For Daily Behaviors, we found 34.5% of Mexican children and adolescents meet Physical Activity recommendations (Grade 3), 48.4% participate in Organized Sports (Grade 5), 35–75.8% engage in Active Play outdoors (Grade 4), 54.1% use Active Transportation (Grade 5), 43.6% spend <2 h in Sedentary Behavior per day (Grade 4), and 65–91% meet Sleep recommendations (Grade 7). Girls have lower physical activity levels and sports participation than boys of the same age. For Physical Fitness, we found 56.2–61.8% of children and adolescents have an adequate body mass index for their age (Grade 6). For Sources of Influence, we found 65–67% of parents engage in physical activity or sports in a week (Grade 7), 32.2–53.3% of basic education schools have a physical education teacher (Grade 6), and 37% of neighborhoods in Mexico have sidewalks with trees (Grade 4). Regarding Government, several policies and programs aimed at improving children physical activity were launched but their impact and allocated implementation budget are unknown (Grade 6).

**Discussion:**

Mexican children and adolescents engage in low levels of movement behaviors and have limited opportunities to perform such behaviors. The grades and recommendations provided here should be considered to improve such opportunities.

## 1 Introduction

According to the Global Status Report on Physical Activity 2022, around 81% of children aged 11–17 do not meet World Health Organization (WHO) physical activity recommendations (1). The WHO Global Action Plan on Physical Activity 2018–2030 aims to reduce this prevalence and one strategy focuses on strengthening physical activity surveillance across the lifespan (2). The Active Healthy Kids Global Alliance (AHKGA) has made a significant contribution to this endeavor worldwide (3). The alliance developed a standardized global surveillance system focused on evaluating the prevalence of movement behaviors among children and adolescents and the opportunities available to perform these (4). This global survelliance system employs a movement behavior paradigm, where behaviors that contribute to children and adolescent movement are examined separately: physical activity, sleep, active play, active transportation, and sedentary behavior (5). As opposed to only focusing on physical activity, the movement behavior paradigm offers a more granular evaluation of movement among children and adolescents, while identifying several intervention points.

The AHKGA evaluation results are presented in the Global Matrix of Report Cards on Physical Activity for Children and Adolescents (6). For the Global Matrix, a standardized evaluation methodology is followed where several countries develop Report Cards in which grades are assigned to movement behavior-related indicators. Four Global Matrices have been completed to date; the latest one in 2022, where 57 countries from six continents completed Report Cards (4, 7–9). As part of the Global Matrix, Mexico has developed four Report Cards that have improved the surveillance of movement behaviors in Mexican children and adolescents (10–13). This manuscript reports results from the 2022 edition of the Mexican Report Card which aims to provide an up-to-date evaluation of movement behaviors among Mexican children and adolescents and to inform programs and policies to improve these behaviors. This is particularly important after the negative impact the COVID-19 pandemic lockdown had on physical activity and sedentary behaviors in Mexican children and adolescents (14).

## 2 Methods

The Mexican Report Card is a national surveillance system that qualitatively assesses secondary data to monitor movement behaviors among children and adolescents, as well as the opportunities families, communities, and government offer to perform these behaviors. The development of the Mexican Report Card follows three main steps: (1) identification of data sources for each indicator; (2) comparison of the data gathered against established benchmarks; and (3) grade assignment. The 2022 edition of the Mexican Report Card evaluated 11 indicators grouped into four categories presented in Table 1.

**Table 1:** Indicators and benchmarks used in the 2022 Mexican report card.

Indicator	Benchmark
***Daily behaviors***	
Physical activity	% of children and adolescents that accumulate at least 60 min of moderate to vigorous intensity physical activity per day.
Organized sport and physical activity	% of children and adolescents who participate in organized sports and/or physical activity programs.
Active play	% of children and adolescents who engage in unstructured/unorganized active play at any intensity for more than 2 h a day.
Active transportation	% of children and adolescents who use active transportation to get to and from places (e.g., school, park, mall, friend’s house).
Sedentary behaviors	% of children and adolescents who spend no more than 2 h of recreational screen time per day
Sleep	% of children and adolescents who meet Canadian sleep recommendations: 9-11 h a day for girls and boys 5-13 years and 8-10 h a day for adolescents 14-17 years old
Physical fitness*	% of children and adolescents have a body mass index that is adequate for their sex and age.
***Sources of Influence***	
Family	% of parents who meet the Global Recommendations on Physical Activity for Health, which recommend that adults accumulate at least 150 min of moderate-intensity aerobic physical activity throughout the week or do at least 75 min of vigorous-intensity aerobic physical activity throughout the week or an equivalent combination of moderate- and vigorous-intensity activity throughout the week.
School	% of schools where most students are taught by a physical education specialist. % of schools with students who have regular access to facilities and equipment that support physical activity (e.g., gymnasium, outdoor playgrounds, sporting fields, multipurpose space for physical activity, equipment in good condition).
Community	% of communities/municipalities that report they have the infrastructure (e.g., sidewalks, trails, paths, bike lanes) specifically geared toward promoting physical activity. % of parents who report living in a safe neighborhood where they can be physically active.
***Government***	Evidence of leadership and commitment in providing physical activity opportunities for all children and adolescents. Allocated funds and resources for the implementation of physical activity promotion strategies and initiatives for all children and adolescents. Demonstrated progress through the key stages of public policy making (e.g., policy agenda, policy formation, policy implementation, policy evaluation, and decisions about the future).

*Physical fitness also includes the evaluation of cardiorespiratory fitness, muscle strength, muscle endurance, and flexibility. However, since no national data was found to assess these, only body mass index was considered for the grade assessment.

The primary data sources for this evaluation included nationally representative surveys, census, and government documents. To assess Daily Behaviors, we reviewed data from the National Health and Nutrition Survey (ENSANUT) 2016 (15), 2018 (16), the Intercensal Survey 2020 INEGI (17), and the Profile of Upper Secondary Education Students 2019 (18). For Physical Fitness data, we used the 2020 COVID-19 ENSANUT (19). Given the different methods employed in these sources, sample sizes for these indicators significantly vary.

To assess Sources of Influence, we consulted the 2014 National Survey on the Use of Free Time (20), the National Education System 2019–2020 Report (21), The Census of Schools, Teachers and Students of Basic and Special Education (22), the 2020 Population and Housing Census (23), the 2020 National Survey of Victimization and Public Safety Perception (24), and the Sports Practice and Physical Exercise Module 2020 (25). To assess the Government indicator, we examined information from the National Development Plan 2013–2018 (26), the National Strategy for the Prevention and Control of Overweight, Obesity and Diabetes (27), the National Plan for Physical Culture and Sport 2020–2024 (28), the Diet and Physical Activity Action Program (29), the National Commission of Physical Culture and Sports 2020–2024 Institutional Program (30), and the Sectoral Education Program 2020–2024 (31).

If nationally representative data were lacking, research articles published between 2013 and 2021 reporting relevant data on children and adolescents 5–18 years of age were reviewed. When new data were not available to assign a new grade for the 2022 Report Card, the data source and grade from the previous edition of the Report Card (2018) were used. When available, data about the effects of the COVID-19 lockdown on movement behaviors were also examined. The data was gathered between 2020 and 2022.

After data were gathered, the Report Card team met to assign a grade to each indicator. Data obtained were compared against pre-established benchmarks and a grade was assigned based on the proportion of children and adolescents reaching the benchmark (Table 1). The benchmarks and grading system were established and standardized by the AHKGA (6). The assigned grades are based on the Mexican grading system which ranges from 0 to 10, where numbers below 6 represent failing grades and 10 a perfect grade. These grades correspond to the letter grading system other countries use (e.g., 5 = C and 10 = A). During grade assignment, national data took precedence over regional data. Age and gender disparities were also considered; for example, if age or gender differences were observed for a given indicator, the grade was lowered by one point (e.g., from 7 to 6). Grades were assigned as follows:

- 9–10 = we are succeeding with 81–100% of children and adolescents
- 7–8 = we are succeeding with 61–80% of children and adolescents
- 5–6 = we are succeeding with 41–60% of children and adolescents
- 3–4 = we are succeeding with 21–40% of children and adolescents
- 0–2 = we are succeeding with ≤20% of children and adolescents.

## 3 Results

**Figure 1:**
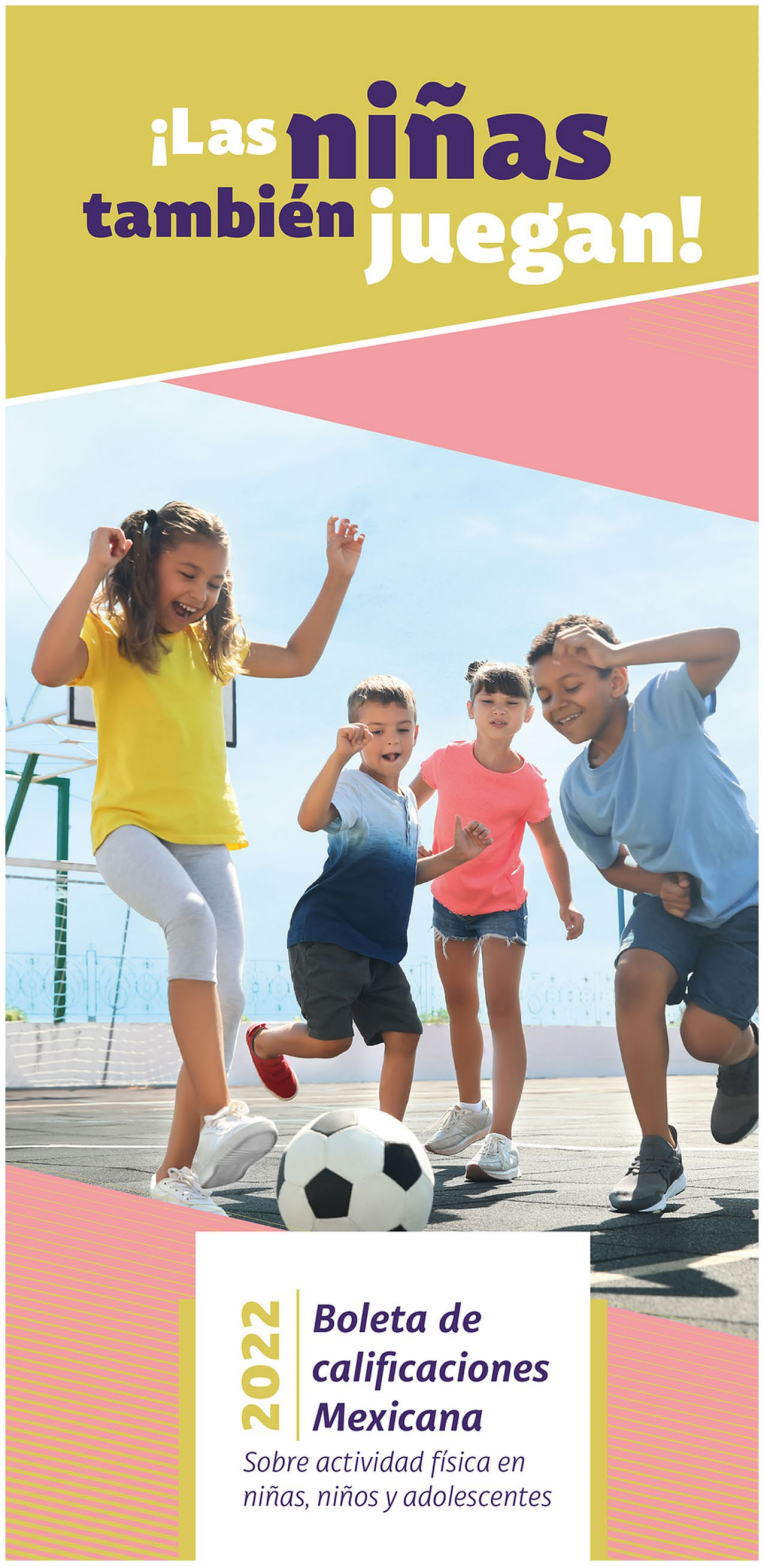
Front cover and theme of Mexico’s 2022 report card.

Of the 11 indicators evaluated in the 2022 Mexican Report Card, 5 obtained approbatory albeit low grades, and 6 obtained failing grades (Table 2). These grades suggest that, except for sleep, movement behaviors are below recommended levels among Mexican children and adolescents, particularly among girls, while opportunities to engage in such behaviors are limited. Hence, the theme and cover of the 2022 Mexican Report Card aims to shed light on this disparity: *Girls get to play too!* This theme highlights the fact that Mexican girls are being left behind and aims to spark actions directed at providing Mexican girls with the same physical activity opportunities boys enjoy (Figure 1).

**Table 2:** Mexico’s 2018 and 2022 report card grades.

Indicator	Rationale	Grade 2022
Overall physical activity	15.4% of children aged 10-14 years and 53.7% of adolescents aged 15-19 years, perform at least 60 min of moderate to vigorous intensity physical activity per day (16).	3
Organized sport participation	48.4% of children aged 10-14 years report having participated in a sport or organized activity in the last 12 months (16).	5
Active play	Children aged 9-12 years spend 2 h a day in active play (32). Children aged 2-5 years spend 3.8 h a day in active play (33). 76% of children aged 6-11 play outdoors at least one day a week (34).	4
Active transportation	54.1% of children over 3 years old walk to school while 1.5% go by bicycle (35). 20.5% of upper secondary students walk to school, 2.1% by bicycle, and 0.13% by skateboard (18).	5
Sedentary behavior	43.6% of children between 10-14 years old spend less than 2 h a day in front of a screen. Adolescents aged 15-19 spend 4.8 h a day sitting (16).	4
Sleep	91% of children aged 10-14 years sleep at least 9 h a day (15). 65% of adolescents aged 15-17 years sleep at least 8 h a day (15).	7
Physical fitness	61.8% of children aged 5-11 years have adequate BMI values for age (19). 56.2% of adolescents of 12-19 years have age-appropriate BMI values (19).	6
Family	65-67% of parents engaged in physical activity or sports in the last week, with an average of 230 min per week (20).	7
School	The percentage of public schools with a physical education teacher is 33.4% in pre-school, 53.3% in primary, and 32.2% in secondary (21). An average of 74.4% of schools have recreational areas and 83.9% have patios (22).	6
Community and environment	37% of the neighborhoods in Mexico have all their streets with sidewalks and trees, 13.4% have public lighting, and 6.5% have a wheelchair ramp (23); 0.5% of the total number of roads in towns with 5,000 or more inhabitants have bicycle paths or cycle lanes (23); 62% of Mexican adults have stopped allowing their children to spend time outside home (24); 60% of the physically active population goes to public facilities (25)	4
Government	Several policies and programs aimed at improving children and physical activity were launched between 2013-2018. However, there is no clear delineation about the responsibilities of the different sectors involved in the implementation of these programs and policies. No considerable progress was identified between 2013-2020 around investments, policies, and programs.	6

### 3.1 Physical activity: 3

Data from ENSANUT 2018 show that only 15.4% of children 10–14 years old and 53.7% of adolescents 15–19 years old accumulate at least 60 min of moderate to vigorous intensity physical activity per day (16). The proportion of boys that meet this recommendation is higher than that of girls in both age groups (55.5% vs. 41.3%, respectively). The proportion is also higher in rural than urban areas in children (rural 17.4% vs. urban 14.7% respectively) and adolescents (rural 48.1% vs. urban 47.5%, respectively). In the ENSANUT COVID 2020 national survey, 68.8% of children aged 10–14 and 60.0% of adolescents aged 15–19 years old reported a decline in the time spent on physical activity or sport (19). Based on the low prevalence of physical activity and the declining trend, a grade of 3 was assigned to this indicator.

### 3.2 Participation in organized physical activity and sport: 5

ENSANUT 2018 reports that 48.6% of children 10–14 years old participated in some form of organized physical activity or sport in the last 12 months (16). Girls report lower participation than boys (41.3% vs. 55.5%). A lower proportion of children in urban areas participate in organized physical activity or sports than children in rural areas (47.1% vs. 52.8%). The current participation of Mexican children and adolescents in organized physical activity and sports earned this indicator a grade of 5.

### 3.3 Active play: 4

There were no national data to evaluate this indicator. Five studies reporting data on active play among Mexican children and adolescents were identified, and the grade for this indicator was based on their data. A study conducted in Morelos reports children aged 9–12 years spend between 1.8–2.3 h a day in active play (32). Another study reports that children aged 2–5 years in Mexico City spend approximately 3.8 h a day in active play (33). Furthermore, a study that included Mestizo and Tarahumara children aged 6–14 years found that 45% of Tarahumara children spend more than 4 h per week in active play compared to 27% of Mestizo children (36). A study among 1,509 children ages 6–11 from Guadalajara, Mexico City, and Puerto Vallarta reports that 76% play outdoors at least 1 day per week (34). Finally, a study of 403 Mexican students 15–20 years old examined the time they spend per day in active play, where 56% were classified as active in their leisure time (37). Together, these studies show that Mexican children and adolescents from different regions in the country spend around 2–4 h a day in active play; thus, a grade of 4 was assigned to this indicator.

### 3.4 Active transportation: 5

Data from the Intercensal Survey 2020 shows that 54.1% of children over 3 years of age walk to school, while 1.8% use a bicycle (35). The 2019 Student Profile Survey from the Mexican Ministry of Education reports that 20.5% of students over 15 years of age commute to school by walking, 2.1% by riding a bike, and 0.13% by skateboarding (18). These data suggest that active transportation is low among Mexican children and adolescents. Hence, a grade of 5 was assigned.

### 3.5 Sedentary behavior: 4

Data from ENSANUT 2018 show that 43.6% of children between 10 and 14 years old spend less than 2 h per day in front of a screen (16). These levels are similar in boys and girls (44% vs. 43.2%, respectively). More children in rural than urban areas meet this recommendation (61.5% vs. 37.2%, respectively). ENSANUT 2018 also reports the time spent sitting per day is higher among 15-19-year-old girls than boys (300 vs. 286 min, respectively). During the COVID-19 lockdown, 41.2% of children aged 10–14 years and 35.5% of adolescents aged 15–19 years reported an increase in the time spent sitting or reclining (19). These levels of sedentary behavior earned this indicator a grade of 4.

### 3.6 Sleep: 7

ENSANUT 2016 data shows that 91% of children aged 10–14 years sleep at least 9 h a day (15), where more girls than boys (95% vs. 87%, respectively) meet this recommendation. This data also shows that 65% of 15 to 17-year-old adolescents sleep at least 8 h per day (15). This data suggests that two-thirds of Mexican children and adolescents are getting the recommended sleep for health, which granted a grade of 7 to this indicator.

### 3.7 Physical fitness: 6

The Physical Fitness indicator includes the assessment of body mass index, cardiorespiratory fitness, muscle strength, muscle endurance, and flexibility. Because national data was only available for body mass index, the grade for this indicator was based on this component. Data from ENSANUT COVID 2020 shows that 61.8% of children 5–11 years have an adequate body mass index (BMI) for their age (60.8% in boys and 62.8% in girls) (19). ENSANUT COVID 2020 data shows that 56.2% of adolescents aged 12–19 years have adequate BMI values for their age (56.9% in boys and 55.4% in girls) (19). More children in rural areas have adequate BMI values than children in urban areas (65.5% vs. 60.7%, respectively). Based on the proportion of Mexican children and adolescents who have an adequate BMI for their age, a grade of 6 was assigned.

### 3.8 Family: 7

Data from the National Free Time Use Survey 2014 shows that between 65 to 67% of parents engage in physical activity. The survey also reports parents accumulate an average of 230 min of physical activity per week (20). Based on this, a grade of 7 was assigned.

### 3.9 School: 6

The National Education System 2019–2020 Report shows that 33.4% of preschools, 53.3% of primary education schools, and 32.2% of secondary education schools have a physical education teacher (21). The 2013 Census of Schools, Teachers, and Students of Basic and Special Education reports that 63.9% of public schools have sports and recreational areas, while 76.8% have patios (22). In private schools, these percentages are higher: 85% have sports and recreational areas, while 91% have patios. With the data available, we could not assess the frequency and duration of the physical education classes that children and adolescents receive at school. We also identified school-based initiatives aimed at promoting physical activity, but we found no data to assess if these were implemented and what effects they achieved. When averaged, the available data show 40% of basic education schools have a physical education teacher, while 79% of schools have spaces for children to engage in physical activities. Thus, a grade of 6 was assigned.

### 3.10 Community and built environment: 4

According to the 2020 Population and Housing Census, 37% of the neighborhoods have sidewalks and trees, 13.4% have public lighting, and 6.5% have ramps for wheelchairs (23). Further, 0.5% of the total number of roads in towns with ≥5,000 inhabitants have bicycle paths or cycle lanes (23). Regarding safety, the 2020 National Survey of Victimization and Perception of Public Security data show that 62% of adults have stopped allowing their children to go outside due to low safety perception (24). Additionally, data from the Sports Practice and Physical Exercise Module 2020 shows that 60% of the physically active population reports going to public facilities to perform physical activities (25). Finally, according to the Health Sector Program 2013–2018, 83 spaces for physical activity have been reactivated and recovered, benefiting 219,427 inhabitants (28). Based on this, a grade of 4 was assigned to this indicator.

### 3.11 Government: 6

Several policies and programs were introduced between 2013 and 2020. The National Development Plan 2013–2018 (26) included an objective aimed at promoting inclusive sports to foster a culture of health. In the Health Sector Program 2013–2018, two strategies aimed to promote physical activity were introduced: the National Strategy for the Prevention and Control of Overweight, Obesity and Diabetes (27) which aimed to promote physical activity in school, work, and community settings, and the Diet and Physical Activity Action Program which aimed to implement 116,904 physical activity promotion events in different settings (29). Regarding physical activity-specific promotion programs, the Health Sector Program 2018 Progress Report indicates that 119,797 actions were carried out to promote physical activity, but it is unclear which ones were directed at children and adolescents (28). Finally, the National Plan for Physical Culture and Sports 2021–2024 has three objectives that seek to promote recreational and competitive sports participation in an inclusive manner (30).

The Education Sector Program 2013–2018 included an objective aimed at placing physical activity and sports participation as an integral component of children education (30). It established actions to strengthen the sports infrastructure in the educational system, promoting physical activity and sports participation in educational institutions, and the promotion of physical activity in extracurricular hours for children and adolescents. Information about the results of these actions is lacking. For the 2020–2024 period, the Education Sector Program established objectives to guarantee the right to physical culture and sports, with an emphasis on integrating schools, social inclusion, and promoting healthy lifestyles in school-age children (31). The program prioritized the promotion of physical activity, play and sports participation in schools and the participation of children from all socioeconomic groups.

Regarding investments, we found no information on the specific budget allocated to physical activity promotion policies and programs in the 2013–2018 government period. According to the Evaluation of Consistency and Results of the Physical Culture and Sports Program in 2017, progress was observed with respect to the percentage of support offered to members of the National Sports System in terms of sports infrastructure, with a value of 111% with respect to a goal of 100%. In contrast, the percentage that municipalities in Mexico allocated to sports participation was 0.18% (28). Regarding other resources, the Annual Program of Physical Culture and Sports 2021 assigned $2,676,498,775.0 Mexican pesos for strategies to promote sports participation (26). Concerning leadership, the government documents and reports reviewed here do not clearly delineate the responsibilities of the different sectors involved in the design and implementation of the programs and policies introduced. In sum, no considerable progress was identified between 2013 and 2020 around investments, policies, and programs aimed at promoting physical activity in Mexican children and adolescents. Hence, a grade of 6 was assigned to this indicator.

## 4 Discussion

The 2022 Mexican Report Card is the most comprehensive evaluation of movement behaviors and related opportunities among Mexican children and adolescents. We evaluated 11 indicators, where five obtained approbatory grades and six obtained failing grades. Except for sleep, all movement behaviors evaluated received failing grades, which indicates Mexican children and adolescents are not meeting the recommended levels for these behaviors, particularly girls. In terms of opportunities, the 2022 Mexican Report Card grades show families, schools, communities, and the government are not providing sufficient opportunities for Mexican children and adolescents to perform health-enhancing movement behaviors. Though these grades suggest Mexico is not on track to meeting the Global Action Plan on Physical Activity 2018–2030 goals (2), they highlight several opportunities for improvement.

### 4.1 Daily behaviors

Except for sleep, all Daily Behaviors evaluated received failing grades, indicating less than half of Mexican children and adolescents are meeting the recommended levels for these behaviors. Girls have lower levels of physical activity and sports participation, than boys of the same age. This has been a recurring finding in each edition of the Mexican Report Card and could speak to gender stereotyping which has been identified as a barrier to physical activity (38). The Mexican culture, traditions, and gender norms dictate girls should move/look certain ways, which limits opportunities for girls to move more (39). Hence, the theme of the 2022 Mexican Report Card – *Girls get to play too –* aims to shed light on this disparity and to spark actions directed at promoting movement behaviors among Mexican girls.

Based on the Global Matrix 4.0 estimates, Mexico’s average grade across daily behaviors indicates between 40 to 46% of children and adolescents are meeting recommended levels for movement behaviors (4). These estimates align with those reported in the Colombian Report Card, and are higher than those reported in the Argentina, Brazil, Chile, and Uruguay Report Cards (4). Finally, the COVID-19 pandemic in Mexico negatively impacted three of the Daily Behaviors evaluated: physical activity and sports participation decreased, while sedentary behavior increased (19). Though this was not penalized in the grades reported here, it echoes what Report Card leaders from the countries participating in the Global Matrix 4.0 reported (4), and calls for efforts to counter the adverse effects the pandemic had on children and adolescent movement.

Compared to grades in the 2018 Mexican Report Card (13), the Sedentary Behavior grade increased from 3 to 4, given that the proportion of girls and boys aged 10–14 years who spend less than 2 h a day in front of a screen rose from 33% reported in ENSANUT 2012 (40) to 44% reported in ENSANUT 2018 (16). For Active Play, the grade increased from 3 to 4 given that more regional data were identified providing a better understanding of this behavior but no tangible improvements were observed. In contrast, the Physical Activity grade dropped from 4 to 3 given that physical activity levels decreased among children from 17.2 to 15.4%, and in adolescents from 61 to 53.7% according to ENSANUT 2016 (15) and 2018 (16), respectively. Active Transportation, Sports Participation, and Sleep grades remained the same as in 2018 because they were based on the same data. At the time this evaluation was completed, no new data was available to update these grades. Although the small grade improvements observed are promising, those that did not change or worsen indicate Mexico is not on track to meeting the Global Action Plan on Physical Activity 2018–2030 goals (2).

### 4.2 Physical fitness

The 2022 Physical Fitness grade shows that around 60% of Mexican children and adolescents have an adequate BMI for their age. The Physical Fitness grade dropped from 7 in the 2018 Mexican Report Card (13) to 6 in the 2022 edition. This is because the percentage of children and adolescents who have an adequate BMI for their age decreased by 5% in children aged 5–11 years, and 7.5% in adolescents aged 12–19 years. This grade highlights the alarming trend of increasing overweight and obesity identified in Mexican children and adolescents (41), and calls for urgent measures to halt it.

No nationally representative Mexican data was found to grade cardiorespiratory fitness on which the Global Matrix grade assessment is based. Hence, we are unable to compare Mexico’s Physical Fitness grade against those assigned by other countries with Report Cards. In fact, this indicator has the most incomplete data across countries in the Global Matrix (4), which underscores the difficulty of evaluating cardiorespiratory at a national level. Because physical fitness is a strong indicator of health in children and adolescents (42), it should be included in national-level surveillance efforts. The 20-meter shuttle run test has been identified as a simple test that can be used in real-world settings (e.g., during physical education class) (43) to assess physical fitness worldwide and should be considered for surveillance in Mexico.

### 4.3 Sources of influence

Of the indicators included in this category, Family obtained the highest grade, showing over 60% of parents in Mexico engage in adequate levels of physical activity (20). The grade for School indicates that 40%-79% of public basic education schools in Mexico have resources and spaces for physical education/activity. The Community and Environment grade indicates Mexico does not offer sufficient or safe opportunities for children and adolescents to be physically active in their community. On average, these grades show between 40 to 46% of children and adolescents in Mexico have physical activity-conducive opportunities/environment in their communities (4). This is higher than the average grades reported by Argentina but lower than those reported by Brazil, Chile, Colombia, and Uruguay in the Global Matrix 4.0 (4).

Most Sources of Influence indicators maintained or increased their grades. The School grade increased from 3 assigned in 2018 (13) to 6 in 2022. This is because national data show an increase in the percentage of public schools with a physical education teacher (from 36.3% reported in the 2018 Report Card to 39.6% in this edition). The Family indicator was graded for the first time in the 2022 Mexican Report Card and sets a useful precedent to monitor the example Mexican parents are setting for their children. The Community and Environment indicator grade in this evaluation was 4 which is the same as that assigned in 2018 (13). This indicates no tangible improvements in community environments for children to engage in physical activities were observed during this period. Overall, these grade changes show little progress has been made since the 2018 Report Card in terms of providing a family, school, and community environment that promotes physical activity.

### 4.4 Government

A grade of 6 was assigned to this indicator because several government policies and programs were introduced between 2013 and 2020 and specific investments were directed at physical activity promotion efforts. However, no considerable progress was identified from the 2018 to the 2022 Mexican Report Cards around these policies and programs. In addition, there is no information about the budget assigned to the implementation of these policies or the impact they are having, other than the number of individuals they reached. In the Global Matrix 4.0, Mexico achieved the same grade as Uruguay, higher grades than Argentina and Brazil, and lower grades than Chile and Colombia (4). In line with conclusions from Report Cards around the world (4), Mexico’s physical activity-related policies were found to have limited comprehensiveness, implementation, and effectiveness.

The 2022 Government indicator grade was 6 which is the same as that assigned in 2018. This suggests that no improvements were observed around policies, strategies, and investments directed at promoting children and adolescent physical activity in that period. The identified strategies and investments introduced by the Mexican government between 2013–2018 seem to be insufficient to positively impact children and adolescent movement behaviors. In addition, physical activity government initiatives remain disconnected and poorly articulated, which limits their implementation likelihood. Mexico is part of the 45% of countries that lack of a national plan for physical activity (44); all the Mexican Report Cards issued to date suggest a national plan that strategically connects all initiatives and clearly delineates implementation responsibilities is needed.

### 4.5 Limitations

This evaluation should be interpreted considering the following limitations. The grades are based on secondary data that was collected using self-reported instruments and, in some cases, using non-validated instruments. Data across age groups is limited; there is no data on movement behaviors in children younger than 10 years, or sports participation data in adolescents older than 14 years. The active transportation data is for children older than 3 years and is not stratified by sex. There is no nationally representative data on Active Play or Physical Fitness in Mexico. Finally, grades for Active Transportation, Sports Participation, and Sleep are based on data that is not up to date; thus, these grades may not reflect the current prevalence of these behaviors.

### 4.6 Recommendations

The Mexican Report Card seeks to inform programs, initiatives, and policies to improve movement behaviors among children and adolescents. An extensive report including detailed recommendations on how to improve each indicator grade has been published elsewhere (45). Here is a summary of the main recommendations derived from the 2022 Report Card grades:

1. Provide opportunities for children and adolescents to move more, particularly for girls. Girls should enjoy the same physical activities and sports boys enjoy and should be motivated to move and play freely. Families have a critical role and should motivate all children to move more.
2. The national surveillance of physical activity, sports participation, active transportation, active play, sedentary behavior, and sleep should be strengthened. National-level data should be collected for all behaviors, for all age groups (0–18 years old), and stratified by sex. Validated questionnaires should be employed for this endeavor.
3. A national surveillance system for physical fitness should be implemented. At a minimum, cardiorespiratory fitness should be assessed using valid, standardized methods. For example, the 20-meter shuttle run test is a simple and cost-effective test that could be implemented at the national level in schools during physical education (43).
4. Schools should offer the statutory hour of physical education per week in preschool and elementary school, and 2 h per week in secondary school (46). The physical education class should be led by a physical education teacher. The recommendations by the national strategy for enhancing high-quality physical education should be followed (47).
5. Investments should be allocated toward rehabilitating existing spaces to perform physical activities in the community (e.g., parks), and toward building a safe infrastructure for walking and cycling.
6. A national physical activity plan that strategically connects all government initiatives and clearly delineates implementation responsibilities should be developed. Initiatives should be evaluated to determine how they are being implemented and what impacts they are having. This is essential to coordinate the implementation and goals of all initiatives and to determine if investments in such initiatives are paying off.

## 5 Conclusion

The 2022 Mexican Report Card grades show that movement behaviors among Mexican children and adolescents are below recommended levels, while opportunities to perform these behaviors are limited. The grades also show Mexican girls are being left behind since they have lower physical activity and sports participation than boys of the same age. The small or no improvements observed in grades from 2018 to 2022 evidence the lack of commitment to address the physical inactivity epidemic among Mexican children. Though results suggest Mexico is not on track to meeting the Global Action Plan on Physical Activity 2018–2030 goals, they highlight several opportunities for improvement. Families, schools, communities, and the government should consider the grades and recommendations provided here to ensure Mexican children and adolescents enjoy the numerous health benefits of a physically active life.

## Data availability statement

The original contributions presented in the study are included in the article/supplementary material, further inquiries can be directed to the corresponding author.

## Author contributions

GA: Data curation, Formal analysis, Investigation, Writing – original draft. JRL: Data curation, Formal analysis, Investigation, Resources, Writing – review & editing. JOB: Data curation, Formal analysis, Investigation, Project administration, Writing – review & editing. AG-G: Data curation, Formal analysis, Investigation, Writing – review & editing. IG-C: Data curation, Formal analysis, Investigation, Writing – review & editing. MFGV: Data curation, Formal analysis, Investigation, Writing – review & editing. AJ: Data curation, Formal analysis, Investigation, Writing – review & editing. EJ: Data curation, Formal analysis, Investigation, Writing – review & editing. CM: Data curation, Formal analysis, Investigation, Writing – review & editing. YSPM: Formal analysis, Investigation, Methodology, Resources, Writing – review & editing. MPR: Data curation, Formal analysis, Investigation, Writing – review & editing. RARP: Data curation, Formal analysis, Investigation, Writing – review & editing. MPRM: Data curation, Formal analysis, Investigation, Writing – review & editing. KIG: Conceptualization, Data curation, Formal analysis, Investigation, Project administration, Resources, Supervision, Writing – original draft.
